# Beyond the façade of generosity—Regional stereotypes within the same national culture influence prosocial behaviors

**DOI:** 10.1371/journal.pone.0250125

**Published:** 2021-05-17

**Authors:** Alin Gavreliuc, Dana Gavreliuc, Alin Semenescu

**Affiliations:** 1 Department of Psychology, West University of Timișoara, Timișoara, Romania; 2 Teacher Training Department, West University of Timișoara, Timișoara, Romania; Bucharest University of Economic Studies, ROMANIA

## Abstract

We analyzed prosocial behaviors in a field experiment (N = 307) conducted in an urban context (Timisoara, Banat region, Romania), starting from a classical Cross-Cultural Psychology research organized in UK and Iran by Collet & O’Shea in 1976. If the evoked study is focused on comparing prosocial behaviors in two very different national cultures (UK vs. Iran), we compared helping strangers strategies within the same national culture in relation to the regional identities of the help-seeking subjects. A behavioral scenario was created by asking naïve participants to offer support and give directions to a place even if they did not know its whereabouts. Drawing on social identity theory, it was tested whether *regional belonging* of the help-seeker (in-group vs. out-group) predicts the availability of help-givers for offering help, their availability for giving wrong directions, as well as their emotional expressiveness. Results are interpreted within the perspective of social distance between groups and show that the more distant regional identities are perceived to be, the less generous help-givers are, both in terms of their decision to help and to give wrong directions, as well as in their expressed emotions.

## General introduction

Prosocial behavior has become widely accepted as an essential resource for ensuring social welfare, both at the (inter)individual and societal level. It is considered a relevant contributor to a better society [1]. Most of the studies focused on identifying individual and cultural/societal predictors of prosocial behavior [1–3], yet a substantial amount of variability has been observed in different interaction contexts [4–6], suggesting that interpersonal and contextual factors remain important areas of research. Within this perspective, the relationship between prosocial behavior and group belonging, investigated by considering different cultural and racial groups, revealed a general propensity to favor the in-group. Nuancing this relation further, the idea of connecting prosocial behavior in everyday interactions with stereotypes towards out-groups articulated in within-culture settings could significantly contribute to a better understanding of social dynamics [7].

We analyzed prosocial behavior in an urban context, guided by the findings of a classical research study in Social and Cross-Cultural Psychology, conducted in 1976 by Peter Collet and Gregory O’Shea, which analyzed how different stereotypes influence helping behavior of Near Easterners and Westerners [8]. The authors observed that, when asked for help by a stranger, Iranians gave more misleading directions towards a non-existing place than their English counterparts. The study provided experimental evidence that such differences could not be explained by Iranians’ greater "mischievousness", but could be traced to differences in value systems. The authors noted that Near Easterners were more concerned with form rather than substance; they sought to *appear helpful*, rather than *be helpful*, as an implicit way of expressing a symbolic rejection of the otherness. Instead of comparing two or more different cultures, the present study tested these patterns of prosocial behaviors towards strangers in an intergroup context based on different regional identities belonging to the same national culture (Romanian). We explored how regional stereotypes influence people’s openness to an unknown person belonging to the stereotyped group. For this purpose, we conducted an experiment in which we suggested to the participants that they are interacting with a person who is in need of help, but comes from a different region than theirs and explored how this realization affected their behavior.

## Prosocial behaviors, stereotyping, and their determinants

Prosocial behavior is described as an interpersonal act between a benefactor and recipient(s) of the action, which a particular society defines as generally beneficial for other people or for the social order [9]. It covers a broad category of actions, from holding the door for a colleague in university, volunteering to help in an organization, working together with team members for achieving the goals of an organization, to offering spontaneous support in an accident occurred in public space. In the scientific literature, prosocial behavior is viewed from multiple perspectives, bringing together the intrapersonal (the study of intrapersonal sources of prosocial tendencies), interpersonal & contextual (the study of helper-recipient dyads in specific contexts of interaction), and the societal perspectives (the study of prosocial behavior influenced by societal factors) [10]. In the present study, we focused on the interpersonal/contextual determinants of prosocial behavior, because of our study’s specificity. The main factors evoked in the literature that influence prosocial behavior at this level of analysis, are the pressure of the situation [11–14], the status of the person who requests help (known vs. unknown person; see [14–17]), the nature of engagement of the person who provides support (informal vs. formal task; see [18–21]) and the presence of ethnic/social class/regional stereotypes. Regarding the influence of ethnic stereotypes on helping behavior, Feldman [22] showed that subjects from Greece, France, and the US answered differently to request for directions, when they were solicited by foreigners or compatriots. He found that Athenians were more available to give accurate directions to foreigners than to compatriots. This was the opposite for Parisians, who were more opened to offer correct information to compatriots, while Bostonians gave equally accurate directions to both compatriots and foreigners. Classical studies in this area of research showed that people are influenced in their behavior by the power of ethnic stereotypes, making them more likely to support victims of their own ethnic group than those of a different one [23–25]. In another study conducted by Hopkins and his collaborators [26], in Great Britain, the authors discovered that the strength of ethnic stereotypes determined the availability for helping others. This tendency appears even in mediated interactions. For example, in an experiment done by phone [27], a solicitor of the same-ethnic or different ethnic group, who had car problems, attempted to reach a service station and dialed the home of the naïve subject and requested him/her to call the service station, because he has spent the last coin. Because the victim’s voice was clearly identifiable as belonging to a particular identity, the ethnical and racial stereotypes influenced the level of support he received (higher if the victim was perceived as part of the in-group and lower in the opposite case). In another experiment employing a computer-mediated interaction through emails, the incidence of help-rejection was higher if the subjects perceived the help-seeker as belonging to a different ethnic group than his/her group [28].

Another relevant factor that influences prosocial behavior is related to social class and its associated stereotypes. A classical study on this topic by Exline [29] suggests that people are differently available for their fellow citizens’ requests. Thus, the reactions of American businessmen to requests for directions by conventional students (with "regular clothes") or "hippy" ones, generated very different responses. The more antinormative in terms of clothing the students were, the less help they received. Similarly, Sissons [30] showed, in a study conducted in a crowded place in London (Paddington Station), that the chance of receiving support to find an unknown destination (Hyde Park Corner) depends on the specificity of the roles assumed by a confederate (either working-class or middle-class person). The less power in terms of symbolic capital the help-seeker was perceived to have and the stronger the class-stereotype was, the less likely it was for people to spend time in concrete interaction or offer smiles to their interaction partner.

Regional identity and its related stereotypes can generate similar patterns in prosocial behavior. Because of the intra-culture regional variation of stereotypes [31], the idea of focusing on one culture to better understand this behavior has produced some relevant findings in the last decades. Nettle, Colléony, and Cockerill [32] have examined neighborhoods in the same town of Newcastle, UK, and compared them in terms of prosocial behavior manifested in "dictator games" [33]. They observed that individuals from deprived areas were less likely to allocate resources to other players, and were characterized by lower self-reported social capital, higher incidence of antisocial behaviors and were less willing to return a lost letter. Another classical study, investigating a quantitative and a qualitative measure of helpfulness in a natural environment, indicated that returning "lost" keys to an owner was more likely to occur in the Midwest area of United States compared to the Far West or West area, which was consistent with the regional stereotype of the Midwesterner as more friendly and helpful towards strangers than people from other regions of US [34]. However, to the knowledge of the authors, there are no studies explicitly investigating how the activation of regional stereotypes in intergroup contexts influences prosocial behavior.

One of the mechanisms that influence prosocial behavior is based on intergroup comparisons and stereotyping [26]. Within this domain, one of the most relevant theories in explaining the cognitive processes involved in stereotyping and intergroup bias is social identity theory (SIT) [35–37]. SIT assumes that people strive to maintain and enhance a positive self-concept, and this self-definition is regularly described in terms of group affiliation. Thus, groups provide their members with a social identity, whether or not those characteristics are true of oneself as an individual. Because the subject’s identity is often confronted with adversities or threats, his/her motivation is to sustain a positive social identity by viewing the in-group in a more favorable light than other out-groups [38]. This mechanism facilitates the emergence of negative attitudes towards the "otherness", activating a stereotypical manner of approaching out-groups and encouraging prejudice towards them [39]. This process is strengthened by social categories that become salient in different contexts of interaction, like a spontaneous meeting in public with representatives of those categories [40]. In-group bias is activated due to a "natural categorization" process and is accentuated by a sense of threat in meeting someone different (such as a "stranger" or non-local subject) as well as by attempting to heighten the subject’s self-esteem by comparing with others favorably. Through this process, individuals form a negative impression of out-group representatives, and this often promotes concrete behaviors. For example, they tend to show more prosocial behaviors towards representatives of their in-group and less towards representatives of out-groups [41, 42]. This in-group favoritism in relation to prosocial behavior has been demonstrated in local settings [33, 43, 44], as well as in cross-national contexts, including on nationally representative samples [45–47].

While the effects proposed by the SIT seem to be consistent across multiple contexts and groups, they were typically observed in intergroup contexts characterized by the interaction of mutually exclusive groups [48]. Nevertheless, group belonging can be described by different degrees of inclusiveness, such that two or more groups can be nested in the same superordinate in-group (e.g. different regional identities within the same national or ethnic group). In these situations, can we expect that perceptions of intergroup differences follow the same processes hypothesized by the SIT, like in the case of conventional in-group–out-group contexts? Zúñiga and Asún [49] and Asún and Zúñiga [50] found that in Chile regional and national identities had an inclusive relationship with each other and concluded that the emergence of strong regional identities does not pose a threat for intergroup relations, as such identities are harmoniously included in the wider national identity of Chilean. A similar result was found by Medrano and Gutiérrez [51], who identified no incompatibility between regional identities in Spain and Spanish national identity, on the one hand, and European identity, on the other. It has also been suggested that a common superordinate group can even alleviate problematic intergroup relations, by categorizing out-group members as fellow members of the same higher order in-group [52, 53]. However, other research conducted on regional identities, showed a tendency for strong identifiers with the less inclusive in-group (i.e. regional belonging) to positively differentiate themselves from the more inclusive national in-group [54]. Such results portray an ambiguous picture, primarily determined by the scarcity of studies conducted on the topic.

Even though SIT accentuates the generalizability of in-group favoritism in intergroup situations, it also recognizes that out-groups are not always treated in the same way. For example, when status differentials are involved, low status groups can deny identification with their own group and desire identification with other valued out-groups [55] or show out-group favoritism [56, 57]. Research shows that group identification, group size and power, threat and individual difference variables also influence the degree of bias towards out-groups (for a review, see [58]). Aside from such moderating variables, studies show that the behavior of individuals and groups is often influenced also by the perceived social distance between them and their interaction partners [59], a finding confirmed by studies showing that intergroup behaviors can improve when perceived social distance is reduced experimentally [60]. However, within the paradigm of SIT, *social distance*, defined as the perceived affinity and nearness between people or groups [61], has rarely been investigated as a moderator of intergroup bias and, to the knowledge of the authors, it was never investigated in naturalistic settings, with natural groups.

Group stereotyping often triggers specific *emotions* as well and this is evidenced in several within culture studies. For instance, Wirtz, van der Pligt and Doosje [62] showed an association between the Muslim stereotype in The Netherlands and emotions such as disgust and pity and observed how such emotions anticipated a higher level of social distancing, while a classical synthesis done by Esses, Hannock and Zanna [63] attested that negative emotions are influential determinants of attitudes towards ethnical and racial groups that are pejoratively stereotyped (such as Afro-Americans). Other studies show that emotions such as anger, fear, contempt [64, 65] or anxiety [66] are triggered by the in-group–out-group dichotomy. Tiedens [67] goes as far as to say that the emotions manifested in intergroup contexts are often negative and that these negative emotions are experienced only towards out-group members. Overall, there is a clear pattern of negative emotions associated with out-groups, which are exacerbated in threatening situations.

Beyond these determinants, other researchers focused on variables such as *gender* and *age* as predictors of prosocial behavior. A large project called "National Altruism Study" conducted in the US on a representative sample found that gender is strongly associated with altruistic values, altruistic behaviors and empathy. Even though women show higher scores in most facets of prosociality compared to men [68], results of studies investigating the influence of gender on prosocial behavior are often contradictory. In the study conducted by Collet and O’Shea [1], men from a collectivistic culture (Iran) spent more time with the help-seeking person (role assumed by a confederate male) than men from an individualistic culture (UK), yet no differences between men and women were found in both cultural samples. Even though most studies indicate that women show an increased level of helping when compared to men [5, 69–72], other studies indicate no gender differences or even an opposite tendency [73–75]. Regarding previous research focused on the relationship between age and prosocial behaviors, results are controversial: some of them attested that prosocial behaviors increase with age [76–79], while others did not find any relation [80] or indicated a relatively stable tendency for adult cohorts [81].

## The contextualization of our research–regional stereotypes and prosocial behavior in Romania

Placed by its geography and history in the space of interference between Central Europe, Eastern Europe, and Balkans, Romania has been formed at the border of three different cultural areas, differentiating and approaching them at the same time, in what can be called *the paradox of belonging* ("to belong to all and none of them alike") [82]. The Carpathian Mountains have divided Romanian provinces and created radically different social, political, religious, and administrative structures, which were subordinated especially to the Turks domination in Wallachia and Oltenia (South), Russian and Poland influence in Moldova (North and East), and Habsburg/Austrian/Hungarian domination in Transylvania and Banat (West) until the late XIX^th^ century or beginning of XXth century. More than that, until the end of the First World War, these provinces were never together in the same influence zone. Despite these significantly different heritages, the homogeneity of Romanian national culture and language is outstanding. One of the most influential Romanian historians from the XX^th^ century, formed in the paradigm of the Annals School, has designated this surprising unity as a "historical miracle and enigma" [83], when compared to the modern historical development of other places, such as German or Italian provinces in the same period. He demonstrated that all Romanians from all these provinces could easily recognize and understand each other, without any linguistical difficulties, even if they lived for centuries in entirely different political and administrative environments. Thus, the idea of a powerful homogeneity in terms of national cultural patterns could be reasonably sustained in contemporary Romania, at least when compared with better studied national cultures from Central and Western Europe [84, 85].

Nevertheless, previous studies in Romania focusing on the content of stereotypes indicated a within culture variation, associated with the distribution of ethnic groups or religious affiliations (both more heterogeneous in Transylvania and Banat and more homogeneous in Wallachia, Oltenia, and Moldova) [31, 86]. The same author(s) showed the existence of a moderate regional variation in the content of stereotypes, even though most of the studies reported a stable structure across cultures [87–89]. For example, in a multicultural area such as Transylvania, an ethnic group such as Hungarians is more positively evaluated, both in terms of warmth and competence, than in other Romanian regions. Thus, the idea of similar content of stereotypes of "Romanians" should be regarded with precautions.

The reference point in our study is the Banatian regional identity, because the city where the study took place (Timisoara) is the capital of the Banat region. The Banat region has some unique features that make it distinctive across Romania. Except the national capital area (Bucharest), the "Western" part of Romania (Banat and Transylvania) is the most developed in terms of economic productivity, level of investments, GDP (PPP)/capita, income job security, standard of living, and sustainable development compared to "Southern" regions (Wallachia or Oltenia) and "Eastern" ones (Moldova) [90–92]. The massive wave of social mobility inside and outside Romania (four million Romanians left the country after the official EU accession in 2007) generated a massive loss of population and produced significant changes in social and economic structures. These developments were accompanied by a growing in-flow of remittances and significant changes on the labor market that caused the reorganization of households, gender relations and changed voting behavior [93–96]. Regularly, the pattern of migration attests that "Eastern" and "Southern" historical provinces (i.e. Moldova and Wallachia) provide more migrants than "Western" ones (especially Banat) which, in many respects, accentuated the regional disparities within the country [97].

In terms of social capital, Romania’s Western region (associated with Banat and Timisoara) is also richer than other provinces. For instance, the interpersonal trust in ordinary people is more pronounced, with 15% higher on average in generalized trust than in the other Romanian regions. The entrepreneurship, civic attitudes or volunteering involvement are also more consistently developed [98–101]. Lastly, the inter-ethnic openness is more generous than in other national regions, especially due to shared common history characterized by positive intercultural exchanges, which were encouraged by political structures in the period of former Habsburg or Austro-Hungarian Empire, until the beginning of the XX^th^ century, or by the Romanian Kingdom, until the middle of XX^th^ century, despite the suffocating pressure of homogenization in the Communist period [102–105]. Thus, in terms of ethnic stereotypes and social distance, interethnic relationships are the most favorable in Banat, compared to all other Romanian provinces. The provinces from "inside of the Carpathians mountains" (Banat and Transylvania) have a more pronounced "degree of multiculturalism", because their ethnic compositions are more heterogenic compared to the "outside Carpathians provinces" that belonged to "the Old Kingdom" (i.e. Oltenia, Wallachia, and Moldavia) [105, 106]. Due to this particular heritage, in the Banat region, the image of the" ethnical other" (e.g. German, Hungarian, Serb, and so on) evaluated through the content and tonality of stereotypes is more favorable than the image of the" regional other" (like Oltenian, Wallachian or Moldavian) [99, 105, 107–109]. This approach is based on redrawing the symbolic border of otherness, using the regional criteria instead of an ethnical one, in the differentiation process between in-group and out-groups [107, 109]. Even if ethnicity is at the core of self-definition [110], the evoked outcomes challenged many ethnocentric assumptions within the domain of self and identity research. They opened up the possibility of examining alternative, more diverse formulations of selfhood [111].

Concretely, previous research investigating the attitudes of Banatians towards regional others, using Bogardus’ social distance scale on a sample of 1057 respondents, revealed that Transylvanians are seen the most favorably (with an index of acceptance-rejection of .992, measured on a scale from -1 to 1, as the difference between the most open attitude towards others and the most rejective one, reported to the total number of the expressed attitudes), followed by Wallachians (.607), Oltenians (.503) and Moldavians (.421) (see [99]). An identical pattern was revealed when Banatians were asked if they would vote for a candidate who originates from these regions. Almost 70% declared they would not vote for a Moldavian, with attitudes progressively improving (in this order) for an Oltenian, Wallachian and Transylvanian candidate [99]. In other analyses focused explicitly on the content of regional stereotypes, it was evidenced for the Banatian subject that Oltenian regional stereotype is described by the most numerous attributes, with the most negative tonality, followed by Wallachian, Moldavian and Transylvanian regional identities, the last one with the most positive tonality [99, 105]. Thus, the stereotypes about different regional identities, measured on representative samples in within-culture settings, indicate a specific "hierarchy" [112] in terms of assessing others [113, 114].

## The present study

Studies focusing on prosocial behavior in Romania are uncommon [115, 116], and were only conducted on adolescents samples. They indicated a positive role of prosocial behavior at home [116] and of religiosity [115] on global prosocial behavior. However, none of these studies reported any influence of regional stereotypes on prosocial behavior.

The literature distinguishes between three major categories of prosocial behavior: helping, altruism, and cooperation [117]. In the present study, we focused on *helping behavior*, defined as an act that produces a concrete benefit to or improves another person’s well-being [9, p. 32]. The present study was conducted in a large urban environment, with one of the highest populational density in Romania, namely the city of Timisoara, the capital of Banat region. The central objective of our study was to examine the different patterns of ordinary people’s helping behavior in a spontaneous dyad-interaction with a help-seeking subject belonging to a specific regional identity within the same national culture. For this purpose, we conducted a field experiment in which a help-seeking subject assuming different regional identities approached unknowing individuals on the street and asked for directions. Because the solicitation for help was about a non-existent street, it offered the help-giver the possibility to act cynically and “send the help-seeker to nowhere”, by giving misleading directions.

Based on SIT, we anticipate that help-seeking subjects belonging to out-groups will receive less help than those belonging to the in-group. Nuancing this behavior further, based on previously reviewed studies, we also expect that this intergroup bias will be moderated by the perceived social distance towards the out-group, with behaviors progressively improving as the social distance towards the out-group decreases. More concretely, we expect that:

*Hypothesis 1*: The greater the social distance towards help-seeker’s regional identity is, the lesser the help-giver’s availability to help will be.
*Hypothesis 2*: The greater the social distance towards help-seeker’s regional identity is, the greater the help-giver’s predisposition to give misleading directions will be.
*Hypothesis 3*: The greater the social distance towards help-seeker’s regional identity is, the more negative the help-giver’s emotions will be.

Because the literature focused on gender and age as predictors of helping behavior revealed contradictory findings, we decided not to formulate any hypotheses and instead to approach these topics in an explorative manner.

### Procedure

The present study (so-called "*Could you help me to get to the destination*?") employed one confederate, a student (male), around 30 years old, without any distinctive features, who asked for directions to an unknown place. As a "stranger" or non-local subject, he expressed his own regional identity by a verbal formula: a salute, followed by a short indication regarding his regional belonging, and a short behavioral cue of being "lost". Each passerby confronted with this interaction could stop for a while or pass without stopping at all. If the pedestrian decided to stop, he/she was asked the way to a non-existent place (i.e. "*Ioan Vodă cel Cumplit*" Street). Ioan Vodă cel Cumplit (John III the Terrible) was one of the relatively well known Romanian medieval voivodes from the XVII^th^ century. Despite his notoriety, it does not exist in Timișoara a street with his name. To eliminate the possibility of unwilling error of the naïve subject, after this sequence, he/she was asked about the way to a prominent and very well-known place in the vicinity, which he or she was likely to know ("*Catedrala*"–the Orthodox Cathedral). The Orthodox Cathedral’s monumental building in Timisoara is a symbolic center of the city because, as many native inhabitants have told, "all the streets are flowing to the Cathedral." Similar experimental locations were chosen, regularly placed in a cross-street vicinity, relatively close to the Cathedral (less than 1.5 km on Google Maps, in a direct line). The confederate, coming from a neighboring region of Banat (Transylvania), was the same in all interactions. Each time, the confederate addressed his request by using specific, regional linguistic stereotypical cues, especially in terms of distinctive regional accent, which is very easily recognized in ordinary contexts (see the S1 File for details). For each major Romanian historical region (Banat, Transylvania, Oltenia, Wallachia, and Moldova), a small representative town was designated, which was assumed by the help-seeker as his city of origin. There were: Buziaș (from Banat), Brad (from Transylvania, or "Ardeal"), Băilești (from Oltenia), Giurgiu (from Wallachia), and Vaslui (from Moldavia). A group of seven experts formed by sociologists and geographers designated the relevant small cities from each traditional Romanian region, from a list of 10 towns per region with similar features (in terms of population, cultural significance, and economic level). The factual statements were the following: "Hello. I’m from / I’m coming from… (he mentioned the city)" (step 1a). After a short sequence of non-verbal behavior expressing the fact that he is "lost" (around 5 seconds) (step 1b), he followed with the next sentence: "… and I’m looking for the *Ioan Vodă cel Cumplit* Street. Could you direct me to get to it?" (step 1c). If the pedestrian stopped and listened to the help-seeker (step 1a & step 1b), we considered this behavior as "acceptance of helping others." If the pedestrian indicated the wrong direction to the help-seeker (step 1c), we considered this behavior as "sending others to nowhere." After no more than 10 seconds after indicating (or not) the fictional place, the second statement followed: "And please, could you also show me how to get to the *Catedrala*?" (step 2). Only if the naïve pedestrians answered correctly to the second question, their behavior was taken into account. In this way, we eliminated the possibility of misunderstandings or unwilling errors, like in the referential study conducted by Collet and O’Shea [1]. Whatever the behavior of the subject who accepted the interaction was, in the final stage, a collaborator of the experimenters politely approached the naïve subject, presented him/herself, and briefly described the consumed scenario as part of a field experiment and explained the scientific stake of the research. After that, the collaborator asked for consent to use participant’s data and asked for their age. The collaborator also expressed his/her availability for offering support for any concerns or questions that the pedestrian might have.

### Variables

The independent variable (IV) was the *regional belonging* of the help-seeker (manipulated, with five conditions). The dependent variables were: *acceptance of helping* others operationalized by engaging or not in a conversation with the help-seeker and indicating the availability to help, (DV1); *sending other to nowhere*, operationalized by giving directions to a fictional place or not (DV2); *emotional expressiveness* of the pedestrians, which was measured by a group of experts situated in the location of this spontaneous interaction (DV3), who observed all the communication between the confederate and the naïve subject and assessed his/her emotional disclosure by using behavioral descriptors. *Social distance* towards different out-groups was not directly measured, but retrieved from previous studies investigating the same groups, as measuring it would have complicated the procedure.

Resuming the scenario, the episode began with the confederate approaching a pedestrian who was walking alone, with a request for help. The pedestrian should walk in a “normal” manner, without expressing any manifest rush. The confederate declared his regional identity and suggested his need for help. "Acceptance of helping" (DV1) was defined as the availability of the pedestrian for stopping for a while and responding to the behavior of the help-seeker who was suggesting he is "lost." "Sending other to nowhere" (DV2) was defined as the pedestrian’s behavior of indicating the wrong direction to the solicitor, even if the place doesn’t exist. "Emotional expressiveness" (DV3) was defined as the disclosure of pedestrians’ emotions activated during the whole sequence of the interaction. A group of four experts (psychologists), trained in prior sessions, assessed each naïve participant’s emotional expressiveness. This group was placed in the vicinity of the concrete interaction and seemed to be a group of friends who spontaneously met on the street and talked to each other. They carefully observed the emotional disclosure of the naïve subject using a grid of behavioral descriptors previously created, with a scale between -3 (extreme rejection, recognized by very aggressive behavior, like swearing at the solicitor, spitting in front of him, addressing very unpolite words, threateningly gesticulating, frowning) and + 3 (extreme kindness, recognized by very generous openness, supportive non-verbal behavior, addressing very polite words, gesticulating in a supportive way, smiling), with 0 as neutral openness (impersonal, "dry" behavior). The group of experts was previously trained in working with this scale on at least ten similar interactions before they started being involved in the experiment’s concrete trials. Also, each expert was trained to record on her/his device (a mobile phone) the participant’s score after monitoring the interaction between the confederate and the pedestrian. Each expert recorded her/his evaluation without knowing the assessment of the others.

The dependent variable called "emotional expressiveness" (DV3) was calculated as the mean of all scores given by the group of experts. In assuring the validity of this measurement, we computed the inter-rater agreement (IRA) between evaluators [118, 119] and preserved only the assessments above the threshold of r_WG =_ 0.8. Only three observations had a score below the threshold r_WG =_ 0.8 and were excluded from the analysis.

### The pilot study

Before the actual study was carried out, a pilot study was conducted that included all the mentioned steps (including debriefing), to ensure that all the actors involved in the interaction (naïve subject, confederate) have accurately understood each step of the procedure and the concrete requirements associated with their role. We especially wanted to verify if the naïve subjects adequately understood the "presentation statement" in terms of regional belonging. All naïve subjects involved in the pilot study (N = 18) correctly and univocally recognized the assumed regional identity of the confederate (three times Banatian, Transylvanian and Oltenian, four times Wallachian and five times Moldavian). For this reason, we could reasonably consider that regional stereotypes have become salient in this context of interaction and decided not to carry out a complete manipulation check in the actual experiment, as it would have complicated the procedure. At the same time, all of the naïve subjects have non-ambiguously recognized the help-seeker "as a lost" person "in the middle of the city." Therefore, we decided to preserve all the evoked sequences in the actual experiment.

### Participants and the selection procedure

The experiment took place during the weekdays’ business afternoon hours, in similar locations (described before) and only in regular weather conditions, avoiding any unpleasant atmospheric conditions (like rainy or windy moments). To ensure random sampling of respondents, the confederate started by approaching each 10^th^ person who passed him and who met the criteria for acceptance (to be alone, walking "normally," without hurry) on the street’s established place in every new trial. For each 10^th^ pedestrian who did not fit the description (for instance, he or she was not alone or was in an obvious hurry), the next appropriate pedestrian was approached.

For an adequate calibration of our sample size, we performed a power analyses (PA), using G*Power, version 3.1.9.7 [120]. Because our analyses imply chi-square tests and one-way ANOVA, we performed PA for both. Thus, for an average effect of Cohen’s w = .30, an a priori PA for a chi-square test with df = 4, α = .05 and a power of .80, revealed that the required sample size was N = 133, while if the effect was lower (w = .20), the required sample size was N = 299. Regarding the PA for ANOVA test, for an average effect size f = .25, with five groups, α = .05 and a desired power of .80, the total required sample size was N = 200, while for a smaller effect size f = .20, the required sample size was N = 305. In the end, we decided to select the most conservative option for being assured that our sample size is sufficient to identify even a smaller effect [121]. Based on this rationale, our global sample was established at N > 305.

#### Ethics

The present research was ethically approved by the Scientific Committee of the Center for Social Diagnosis from the Faculty of Sociology and Psychology from the West University of Timisoara. In requesting approval, we described the global research design for the field experiment (title, procedures applied, ethical implications for human subjects, methods, expected results). Even if the pedestrians were not aware of their initial participation in this experiment, their privacy was respected during and after the interaction. All naïve subjects who accepted the interaction with the confederate were debriefed at the end and asked for their consent. Before starting the concrete experiment, we tested, in the already mentioned pilot study, whether similar participants (naïve pedestrians) are likely to be distressed by the proposed scenario. None of them reported any distress once they discovered the research’s true nature at the debriefing stage. More than that, none of the subjects involved in the field experiment (N = 307) reported distress caused by their participation in the experiment.

## Results

SPPS v 21.0 was used to conduct all analyses. The final sample was comprised of 307 individuals (49% female), aged between 18 and 78 years (*M* = 45.64, *SD* = 14.27). A binomial test on the global sample (N = 307) indicated that the proportion of subjects who accepted the interaction and showed "their availability to help" (p = .78), was significantly higher than expected by chance (p = .50), *p* < .01. Therefore, there is a significant propensity to accept the interaction and expresses the availability to help a stranger/non-local subject.

To test our first hypothesis, we first performed chi-square tests to verify whether the acceptance of helping others differs depending on the regional belonging of the help-seeker and its associated stereotypes. Test results revealed a significant propensity to favor the in-group, *χ*^*2*^(1) = 8.595, *p* = .003, *V* = .167, as well as significant differences in the acceptance of helping out-group members, depending on the help-seeker’s regional belonging, *χ*^*2*^(4) = 27.73, *p* < .001, *V* = .30 (see Table 1).

**Table 1:** Cross tabulation of regional belonging of help-seeker and acceptance of helping.

Acceptance of helping (DV1)	Regional belonging of the help-seeker (IV)						*χ*^*2*^	df	*V*
	Banat	Transylvania	Wallachia	Oltenia	Moldavia	Total			
Refused to help	5	6	18	25	14	68	27.73**	4	0.30
Accepted to help	56	59	40	37	47	239			
Total	61	65	58	62	61				

*Note* **p < 0.01, *V =* effect size (Cramer’s V coefficient)

The highest rate of helping was obtained when the help-seeker belonged to the in-group (Banat, 91.8%), followed by the "neighbor" regional identity (Transylvania, 90.8%), while the lowest rates of helping were for Wallachia (69.0%) and Oltenia (59.7%), both regions being situated in the South of the country. The influence of regional belonging of the help-seeker had an effect on helping behavior of *V* = .30 which, according to Cohen’s [122, p. 222] guidelines, represents a large effect. The rate of helping for the in-group was significantly higher than for Wallachians (*Z* = 3.149, *p* < .001), Oltenians (*Z* = 4.145, *p* < .001) or Moldavians (*Z* = 2.253, *p* = .012), but not significantly higher than for Transylvanians (*Z* = .199, *p* = .421). The rate of help for Moldavians was significantly higher than for Oltenians (*Z* = 2.061, *p* = .039), while Transylvanians received significantly more help than all other out-groups. Our first hypothesis (H1) was thus partly supported by the data.

To test our second hypothesis, we used only the subsample of subjects who accepted the initial interaction and expressed their availability to help (N = 239). We initially performed a binomial test, which indicated that the proportion of subjects who did not give misleading directions (p = .89) was significantly higher than expected by chance (p = .50), *p* < .01. Therefore, there is a significant propensity to avoid giving wrong directions. To test whether giving misleading directions is associated with help-seeker’s regional belonging, we conducted two chi-square tests. Results revealed again an inclination to favor the in-group *χ*^*2*^(1) = 6.237, *p* = .013, *V* = .162, as well as significant differences in giving wrong directions, depending on the regional belonging of the help-seeker, *χ*^*2*^(4) = 17.678, *p* = .001, *V* = .27 (see Table 2). The effect of regional belonging *V* = .27 is, according to Cohen ([122, p. 222]), a large one. In-group members were misled significantly less than Oltenians (*Z* = -3.429, *p* < .001), Wallachians (*Z* = -2.743, *p* = .003) or Moldavians (*Z* = -2.472, *p* = .007), but there was no difference between the in-group and Transylvanians (*Z* = -.537, *p* = .295). Regarding out-groups, there were no significant differences between them, with the exception of Transylvanians, who were misled significantly less than all others. Therefore, our second hypothesis (H2) was also partly supported by the data. Like in the previous result, even if the general tendency is to activate an honest and supportive behavior towards the solicitor, the closer the identity of the help-seeker to the South of the country is, the higher the rate in giving wrong directions is (17.5%, if the regional belonging of help-seeker is associated with Wallachia and 24.3% if it is associated with Oltenia), while for the in-group and for Transylvanians these rates are relatively low (1.8% and 3.4%, respectively).

**Table 2:** Cross tabulation of regional belonging of help-seeker and giving misleading directions (N_1_ = 239).

Giving misleading directions (DV2)	Regional belonging of the help-seeker (IV)						*χ*^*2*^	df	*V*
	Banat	Transylvania	Wallachia	Oltenia	Moldavia	Total			
Gave misleading directions	1	2	7	9	7	26	17.68**	4	0.27
Did not give misleading directions	55	57	33	28	40	213			
Total	56	59	40	37	47				

*Note* **p < 0.01, *V =* effect size (Cramer’s V coefficient)

Regarding the specificity of the emotional expressiveness in relation to the regional belonging of the help-seeker, Table 3 presents the means and standard deviations for this indicator in all cases.

**Table 3:** Means and standard deviations for emotional expressiveness as a function of regional belonging of help-seeker.

Regional belonging of the help-seeker	Subsample of subjects who refused to help			Subsample of subjects who accepted to help			Global sample		
	(N_1_ = 67)			(N_2_ = 237)			(N = 304)		
	*N*	*M*	*SD*	*N*	*M*	*SD*	*N*	*M*	*SD*
Banat	5	0.15	0.65	56	2.17	0.66	61	2.00	0.86
Transylvania	5	-0.25	0.53	59	1.78	0.68	64	1.62	0.87
Wallachia	18	-0.87	1.04	39	1.12	1.04	57	0.49	1.39
Oltenia	25	-1.13	1.11	37	0.98	0.73	62	0.05	1.45
Moldavia	14	-0.79	0.76	46	1.34	0.80	60	0.84	1.20
Total	67	-0.90	1.03	237	1.55	0.88	304	1.01	1.37

**Fig 1:**
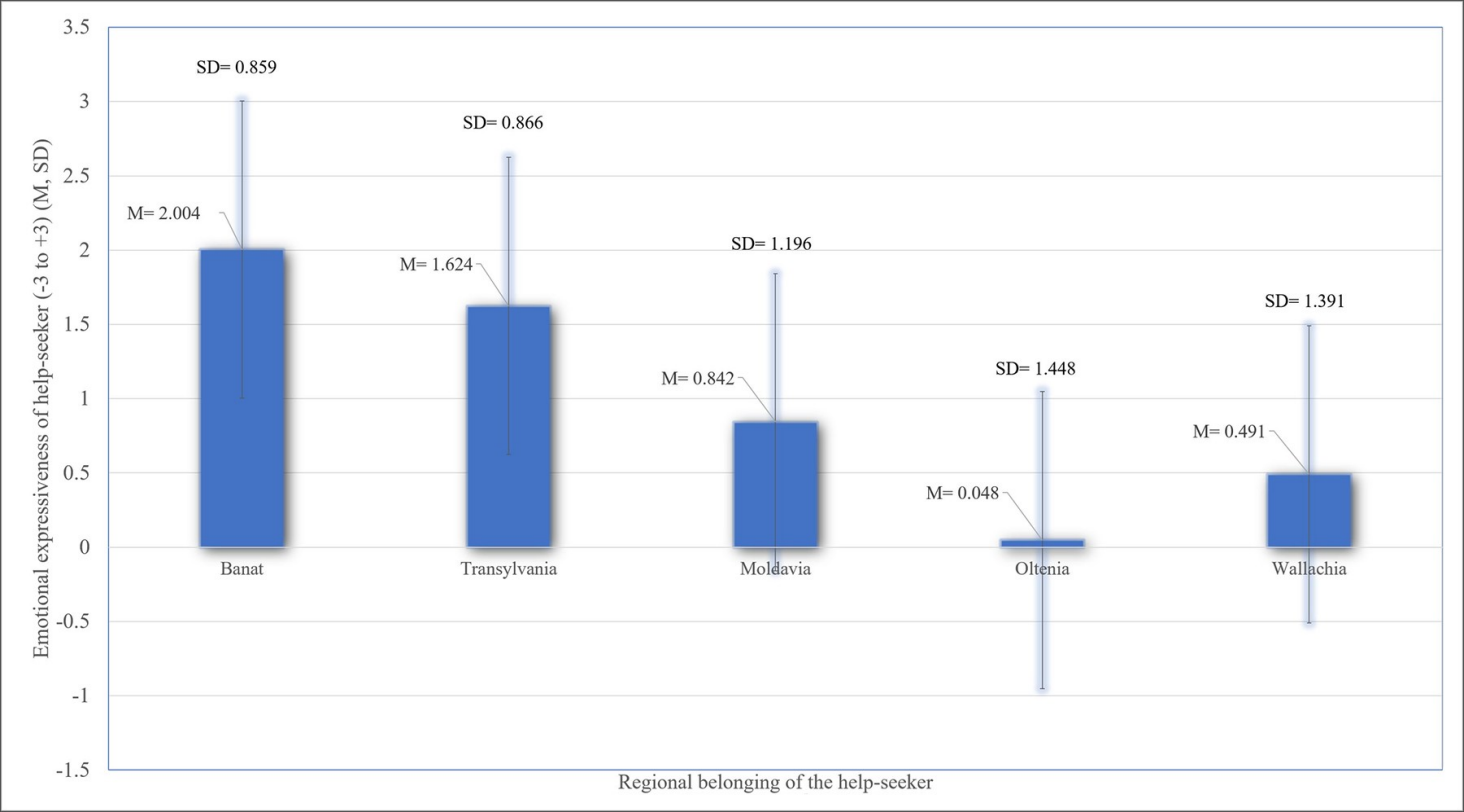
Association between emotional expressiveness of the help-seekers and regional belonging of the help-seekers. Fig 1 shows the relationship between regional belonging of the help-seekers (Banat, Transylvania, Moldavia, Oltenia, and Wallachia) and the emotional expressiveness of the help-seekers on a behavioral descriptors scale (from -3 to +3), where the large blue bar represents the mean score (M) of this variable, and the thin bar represents the standard deviation (SD) of it.

As indicated in Table 3, the emotional expressiveness was more negative when the help-seeker originated from problematic regions, like Wallachia, Oltenia and Moldavia (see Fig 1). To test our third hypothesis, we first conducted one-way ANOVAs to investigate whether there were any differences in the subjects’ emotional expressiveness depending on the help-seeker’s regional belonging. There were significant differences between the in-group and out-group, *F*(1, 302) = 45.799, *p* < .001, *η*_*p*_^*2*^ = .132, and the effect of regional belonging was significant for the global sample (N = 304), *F*(4, 299) = 28.738, *p* < .001, *η*_*p*_^*2*^ = .273, evidencing a large effect (see [122]), but also for the sub-sample of subjects who refused to interact with the help-seeker (N_1_ = 67), *F*(4, 62) = 3.322, *p* = .016, *η*_*p*_^*2*^ = .176 (large effect) and for the sub-sample of subjects who accepted to interact with the help-seeker and expressed their “availability for helping” (N_2_ = 237), *F*(4, 232) = 19.066, *p* < .001, *η*_*p*_^*2*^ = .247 (large effect).

In order to verify where the significant differences in terms of emotional expressiveness were, we conducted multiple comparisons with Tukey correction on the global sample (N = 304). Results revealed significant differences between the in-group and other regional identities, like Moldavia (*t* = 5.52, *p* < .001, mean difference [Banat–Moldavia] = 1.16), Wallachia (*t* = 6.86, *p* < .001, mean difference [Banat -Wallachia] = 1.51) and Oltenia (*t* = 9.33, *p* < .001, mean difference [Banat-Oltenia] = 1.95), while there was no significant difference between the emotional expressiveness towards in-group members and Transylvanians. Regarding comparisons between out-groups, the emotional expressiveness towards Transylvanians was significantly more positive than towards all other out-groups, while the emotional reactions towards Oltenians were significantly more negative than towards Moldavians; no difference between the emotional reactions towards Oltenians and towards Walachians was observed. The same patterns of differences could be observed in both subsamples: Those who refused the interaction with the help-seeker (N_1_ = 67) and those who accepted the interaction with the help-seeker (N_2_ = 237). Therefore, our third hypothesis (H3) was, again, partly supported by the data.

Based on other research suggesting that emotions can mediate the relation between categorization and intergroup bias [123, 124], we conducted two binary logistic regressions to investigate the mediating effect of emotional expressiveness in the relation between regional categorization and acceptance of helping, on the one hand, and the relation between regional categorization and giving misleading directions, on the other. For these analyses, we fist dummy coded regional belonging with Banatians as the reference group. When regional belonging was added as the only predictor, it significantly predicted helping behavior, *χ*^*2*^(4) = 28.890, *p* < .001, Nagelkerke *R*^*2*^ = .138, the pattern of differences being identical to the one previously described (i.e. the in-group receiving significantly more help than all other groups with the exception of Transylvanians). However, when emotional expressiveness was also introduced in the model, the variance explained rose (Nagelkerke *R*^*2*^ = .711) and only emotional expressiveness significantly predicted helping behavior *χ*^*2*^(1) = 64.527, *p* < .001, *OR* = 9.091, 95% CI [5.305, 15.577], suggesting full mediation. Our analysis indicates that the odds of offering help are between 5.3 and 15.5 times higher when emotional expressiveness towards the help-seeker increases by one unit. The same analysis was conducted with “sending other to nowhere” as DV, revealing the same pattern of results. Adding emotional expressiveness to the model increased Nagelkerke *R*^*2*^ from .155 to .296 and made non-significant all differences caused by regional belonging, suggesting again full mediation by emotional expressiveness. The impact of emotional expressiveness remained significant in the full model, *χ*^*2*^(1) = 16.364, *p* < .001, *OR* = 3.120, 95% CI [1.798, 5.414], indicating that the odds of not giving misleading directions are between 1.8 and 5.4 higher when emotional expressiveness improves by one unit. Therefore, our analyses suggest that categorization may trigger specific emotions that, in turn, can affect both people’s availability for offering help as well as their misleading behavior.

Although age and gender influences on helping behavior were not a major focus of our study, we tested their impact on our DVs in an exploratory manner. To investigate the effects of these variables on helping behavior (DV1), chi-square tests of independence were performed. For doing that, age was recoded (from a continuous variable) into a discrete variable with 4 groups (18–35 years old = 90 subjects, 36–50 years old = 108 subjects, 51–65 years old = 95 subjects, +65 years old = 14 subjects). Results of the tests revealed no effect of age, *χ*^*2*^(3) = 5.52, *p* = .138, *V* = .134, or gender, *χ*^2^(1) = .49, *p* = .483, *V =* .04, on helping behavior.

Likewise, the same tests were performed on the other DVs. There was no relation between giving false directions (DV2) and age: *χ*^*2*^(3) = .435, *p* = .933, *V* = .043, or gender *χ*^*2*^(1) = .384, *p* = .536, *V* = .040. Similarly, examining the effect of age and gender on emotional expressiveness (DV3) in all three samples (N = 304 / N_1_ = 237 / N_2_ = 67), revealed no significant effects of age on emotional expressiveness for the whole sample, *F*(3, 300) = 1.101, *p* = .349, *η*_*p*_^*2*^ = .011, for the subsample of subjects who accepted the interaction with others, *F*(3, 233) = .647, *p* = .586, *η*_*p*_^*2*^ = .008, or for the subsample of subjects who refused the interaction, *F*(3, 63) = .286, *p* = .835, *η*_*p*_^*2*^ = .013. In order to investigate the potential differences in emotional expressiveness due to gender, we performed an independent samples t-tests for the whole sample, *t*(303) = .395, *p* = .694, *d* = .045, for the subsample of subjects who accepted the interaction with others *t* (236) = -.035, *p* = .972, *d* = .005 and for the subsample of subjects who refused the interaction with others *t*(66) = -.083, *p* =. 934, *d* = .02, which revealed no differences in emotional expressiveness that can be attributed to gender.

## Discussion

The present study investigated prosocial behavior through a field experiment conducted in an underrepresented culture, by evaluating participants’ reactions to help requests coming from in-group or out-group members. Based on SIT and the empirical evidence evoked in our study’s contextualization, we expected to observe a progressive reduction in terms of prosocial behavior as the social distance towards the out-group increases. Our results indicate that our expectations were partly met. Overall, when regional identities were activated, a clear pattern of favoring the in-group on all investigated outcomes was observed. In-group members were helped significantly more, were misled significantly less and were treated more positively, compared to out-group members. Nevertheless, participants’ behavior towards the out-groups differed significantly, with more favorable treatment towards socially close out-groups and more unfavorable treatment towards socially distant ones.

Regarding the propensity to interact with the help-seeker and the availability to help, our results suggest an apparent openness to others, orienting participants’ behavior by the implicit rule of generosity. At first glance, we can interpret their behavior as generous because, globally speaking, almost 78% of subjects manifest an availability to help. Yet, in a sample where almost ¼ of participants refuse from the beginning any interaction with someone in difficulty, these findings should be regarded with caution. Most of the ordinary people exposed to the interaction with someone who experienced difficulty (suggesting "being lost") expressed their "availability to help." There is a remarkable "generosity" in their behavior. But, behind this appearance, the activation of regional stereotypes associated with the regional belonging of help-seeker attests that simply knowing that the help-seeker comes from another region significantly diminished this generous openness The difference between Banatians’ availability to help in-group members compared to out-group members was a significant one, generating a medium effect size, *V* = .167 (equivalent to *d* = .339), which is remarkably similar to the effect found by Balliet, Wu and De Dreu [41] in their meta-analysis about in-group favoritism in cooperation, conducted on 125 samples, in which they found an average effect of *d* = .32. Concerning our second DV (i.e. sending other to nowhere), a similar pattern was observed: Banatians treated in-group members more favorably that out-group members. The effect was medium in size and similar to the previous one, *V* = .162 (equivalent to *d* = .327). Thus, our findings indicate a decreased availability to help (DV1) and an increased deceiving’ behavior towards the help-seeker (DV2), when he belonged to the out-group.

As our analyses suggest, there is also a significant difference in how different out-groups are being treated, with more favorable behaviors being displayed towards socially close out-groups (i.e. Transylvania) and more unfavorable towards socially distant ones (i.e. Oltenia, Moldavia and Wallachia). In fact, Transylvanians were treated no differently than in-group members on all our measurements, suggesting that in-group favoritism can disappear in relation to groups that are perceived as socially close. Results suggest that manifested emotions mediated this effect: Problematic regional belongings triggered a more negative emotional response of the help-givers, which in turn affected their availability to offer help and their susceptibility in giving misleading directions. Our results are consistent with previous studies using self-reported measures, which showed that in Banat, the stronger the stereotypes of Southeast regional identities are activated (i.e. Oltenia and Wallachia), the higher is the rate of rejection of otherness [99, 105].

One of the most contrasting outcomes indicates a symbolic distancing through what we could have called a *façade generosity*. Even if almost 9/10 of subjects did not voluntary mislead others, when the other was considered to belong to "Oltenian" regional identity, for instance, almost 1/5 activated a cynical behavior and "sent the other to nowhere" (see Table 2). This concrete behavior, accompanied by the negative emotional expressiveness, indicates an increased rate of symbolic rejection of the otherness. Similar to the referential study of Collet and O’Shea [1], the more distant "the other" is perceived, the more the behavior "appears to be helpful" rather than "being helpful." The synthesis of Maass and Arcuri [125] attests a similar pattern. When the help-seeker was perceived as a member of the in-group, this tendency almost disappeared on all three dependent variables (i.e. acceptance of helping others, sending others to nowhere, emotional expressiveness of the pedestrians). Therefore, it is not a matter of inherent "generosity" but suggests that generosity is drastically influenced by the stereotypes activated in people’s minds.

The behavior of help-givers became more negative as the solicitor of help was perceived to belong to a "distant" identity [126] and this was activated by the salience of regional identity of the help-seeker. According to SIT [35, 36], this process is shaped by an inter-group comparison and can be understood as a spontaneous strive for maintaining and enhancing a positive self-concept by consolidating the in-group affiliation. Nevertheless, there were significant differences regarding the behavior towards regional out-groups and these were partly explained by the existing social distance between the in-group and out-groups. Even though we expected to see a clear in-group favoritism, it seems that in intergroup contexts in which the out-group is perceived as socially close (like Transylvanians in our case), the intergroup bias can disappear altogether. This might be due to redrawing the border of otherness to include also groups that are perceived to be “close” and similar to the in-group. According to our hypotheses, we also expected a progressive worsening in intergroup behaviors as the social distance towards the out-group becomes larger, yet this was not always the case. Moldavians received more help and evoked more positive emotions than Oltenians, even though Moldavians are perceived as more distant by Banatians (the in-group), indicating that other moderating variables that were not investigated in the present study might come into play in explaining this pattern of results. Nevertheless, a preferential treatment towards the in-group was evident in comparison to most out-groups, indicating that mutual belonging to a superordinate category (i.e. Romanians) was not enough to mitigate in-group bias. A possible explanation might be that regional identities from the South and East of the country are seen by Banatians as non-prototypical for Romanian identity, undermining in this way group homogeneity and cohesion [127].

Coming from a region where the level of interpersonal trust is highest in Romania, such results can indicate the existence of significant distrust and seeing others as a threatening presence that should be avoided. This was also observed in other cultures, where relationships between ethnic and racial groups recorded a tense and problematic history [43]. This outcome is concordant with studies from *Charity index*, which report that in all Balkan areas, the pattern of rejection towards a needing stranger is high (the average score was 30% in Romania, in 2018, on a representative sample) (https://www.cafonline.org/about-us/publications/2018-publications/caf-world-giving-index-2018), as a general expression of social capital deficit [100, 101, 108]. Cultural factors themselves could also be involved in this way of treating others. Even if we predominantly have only global portraits of this kind of dimensions on Romanian national samples, described by high scores on embeddedness [128], intense loose culture, with weak social norms and a high tolerance for deviant behavior [31, 129, 130], high uncertainty avoidance [131], high scores on in-group favoritism [132] or modest emancipative values [133, 134], these factors could be relevantly considered as the source of this type of generally reserved behavior towards others. Where we have partial data from regional areas (regularly, on regional sub-samples), the more "Western" the regional belonging of the subjects in Romania is, the lower the provinces are in embeddedness, uncertainty avoidance or loose culture and the higher they are in emancipative values [99, 100, 135–137]. These tendencies could also be a potential explanation for our outcomes in terms of Banatian’s reserved prosocial behavior, especially when the help-seekers are stereotypically associated with more "Southern" or "Eastern" regional identities.

This societal background of instability is also accentuated by the intergenerational patterns of distrust towards the "regional other" from "South" and "East," acquired in the socialization process in the last decades in Banat, already evoked in previous ethno-psychological and sociological studies [99–101, 138]. This distrust might be a significant contributor to the instrumental relational logic [139, 140], governed by the general background of high social cynicism recorded in Romania [99, 100, 141].

In the present study we used SIT as a theoretical framework. Nevertheless, the pattern of found results is consistent also with other theoretical explanations, such as realistic conflict theory [142, 143] or integrated threat theory (ITT) [144, 145]. Realistic conflict theory suggests that competition for limited resources can lead to conflict between groups, which becomes manifested in intergroup attitudes and behaviors. As previously reported, Banat region is, after the capital region, the most developed part of Romania, and this generates a high influx of local migrants, especially from the South (Oltenia and Wallachia) and East (Moldavia) parts of the country [146–148]. Individuals from these regions are competing with locals on the labor market and this competition can generate behaviors and emotions towards these groups similar to the ones identified in the present study. ITT, on the other hand, suggests that four types of threats can lead to problematic intergroup relations, namely realistic threats (pertaining to the welfare of the in-group, such as economic or political threats), symbolic threats (pertaining to threats to the “way of life” of the in-group, such as differences in values, norms or morals promoted by other groups), negative stereotypes (pertaining to the negative expectations about the behavior of out-group members) and intergroup anxiety (pertaining to the personal feeling of anxiety that in-group members might experience in intergroup situations). Previous research has already identified the existence of negative stereotypes of Banatians towards Oltenians, Wallachians and Moldavians [99, 105] that, together with realistic threats generated by the competition on the labor market with individuals from these regions, could explain Banatians’ reserved behavior towards the members of these groups.

Concerning the effects of age and gender, our findings suggest that these variables are not relevant factors in influencing helping behavior, at least in a culture with Romanian cultural specificity and in an urban context. Our results indicate that the strength of such reactions is beyond gender and age, evidencing a global pattern of rejection in everyday interaction in Romanian public sphere, as an implicit duplicity towards others, possibly acquired as a hidden survival strategy in the centuries of dependence [149]. Described as a "transgenerational acquisition" [150], this duplicity becomes a functional and routinely used way of thinking and acting in an unpredictable social and political environment [151]. Thus, beyond their parochial identities, this way of relating to representatives of different out-groups is still very influential in everyday life, in ordinary interactions [152]. This tendency can be regarded as an expression of *loneliness next to the others*, that describes interpersonal relationships in a country that assumed the "successful" *incarceration model*, as the communist Romanian experience has promoted it [151].

The findings of the present study may be relevant for practitioners and policymakers for conceiving interventions and policies aimed at reducing intergroup bias and improving intergroup relations. A first step to achieve such outcomes is by strengthening the superordinate, common identity, which may encourage group members to identify more at the superordinate level instead of identifying only at the subgroup level [153], the so-called dual identity model [154]. However, for the superordinate category to be successfully adopted, subgroup identities must be salient and non-threatened by the contact situation [155,156], which in some intergroup situations is hard to achieve, especially when groups have strong negative stereotypes about each other. Moreover, in intergroup interactions, perceptions of in-group prototypicality should be inhibited (see [157]) and the salience of the superordinate identity should be promoted, to encourage group cohesion. Our study shows that greater social distance towards out-groups is associated with less help, more cynical behavior and negative emotional expressivity. Therefore, decreasing the perceived social distance between groups can prove to be a fruitful way to promote identification at the superordinate level, an outcome possibly evidenced by the absence of in-group favoritism and the positive emotional expressivity shown towards the out-group perceived to be socially close.

## Limitations and future directions

Our study suffers from several limitations that are worth noting. First of all, as all participants were residents of Timisoara, the heterogeneity in our sample of Banatians was rather low and would be better to be diversified in future studies. Also, belonging to urban areas inside of a region creates specific stereotypes and corresponding behaviors. For example, Ruston [158] reported that assessing four types of asking for help (for the time, for a particular direction, for changing a coin or for the person’s name) in three areas differing in population density (in the city center, suburbs or a small town outside of Toronto or New York) revealed that for each indicator the frequencies of helping decreased as urban density increased (see also [159] and [160]). Thus, similar investigations conducted in small urban areas or even in rural ones would provide a more nuanced understanding of the present results. Also, the nature of the pedestrians’ specific task was very accessible and with a low level of commitment. Another future investigation can manipulate the difficulty and commitment required by participants’ role, to assess the potential variations in helping behavior due to such factors. Alternatively, a study conducted on a generationally representative sample could test more accurately the potential influence of age on helping-others behavior.

Regarding participants’ regional belonging, this was inferred but not measured. We assumed that all approached individuals were Banatians, yet there is a possibility that some of them had different regional belongings, diminishing thus the found effects. However, this does not affect the conclusions of the present study, as effects are likely to be even higher when this aspect is controlled. Likewise, gender and age are considered in our experimental design, but we did not match participants’ gender and age with the confederate ones. Even if this does not affect our conclusions (participants were randomly assigned to conditions), a more refined future design could include this option, to investigate whether rejection patterns towards regional others remain the same or are neutralized by similarities in age and gender between help-seekers and help-givers.

An important limitation lies in the way the variable emotional expressiveness was measured. Even though we took precautions to avoid bias (i.e. multiple coders unaware of study’s conditions or hypotheses and individual coding of the behavior), the distance between the group of observers and the place of interaction was not controlled. Evaluators were instructed to keep their distance, but no specific instructions were given about how large this distance needs to be, making it possible that in some interactions evaluators were made aware of the conversation that took place. Even though they evaluated the help-giver’s emotional expressiveness independently, we cannot deny the possibility for a collective bias, as Banatian’s stereotypes about other regional identities are well-known within the public. Future studies should control this variable my making sure coders are far enough to observe the interaction but not close enough to hear it.

In our study, social distance towards regional others was not directly measured, but only inferred from previous investigations. Future research that includes subjective and objective measures of this variable would also be a relevant change that can increase the relevance of our analysis. Also, creating a design that investigates, in the same model, the impact of both social distance and social status of out-groups on prosocial behavior is likely to nuance further our understanding of how out-groups are being treated, based on the two dimensions of warmth and competence [161]. Furthermore, a more complex design with direct measures of cultural factors and value orientations, which could also assess dimensions such as individualism-collectivism and their interindividual correspondents, independence-interdependence, or uncertainty avoidance, loose culture and embeddedness, analyzed using a multilevel approach (including both personal and societal level factors), would significantly deepen the scientific demarche. Not at least, for future potential replications, our concrete scenario of interaction could be reconfigured following our findings (i.e., eliminating step 2, which seems to be redundant). Simultaneously, the precise patterns of observed results are still interpretable, as more moderators of intergroup bias are likely coming into play when generating such outcomes.

## Conclusions

In our field experiment, the acceptance of helping others (as an explicit prosocial behavior), sending others to nowhere (as a cynical way of helping), and the emotional expressiveness (that also incorporated an unconscious dealing with strangers) are dependent on regional stereotypes towards out-groups that are activated in participants’ minds, in a spontaneous everyday life interaction. Out-groups that are perceived as distant are helped significantly less, are treated with more cynicism and with more negative emotional expressiveness, while out-groups perceived as socially close are treated no differently than the in-group. Our study provides new evidence to the literature of moderators of intergroup bias and new data on the association between social categorization and prosocial behavior in a highly under-represented culture.

## Supporting information

S1 File (DOCX)
