# Supplementary material for: Beyond the façade of generosity—Regional stereotypes within the same national culture influence prosocial behaviors
Source: PLoS One. 2021 May 17;16(5):e0250125. doi: 10.1371/journal.pone.0250125 (PMC8128266; doi:10.1371/journal.pone.0250125)
Supplement: S1 File — (DOCX) [file pone.0250125.s001.docx]

# S1 File

**Extras from the protocol of interaction**

*(the version for confederate)*

(English translation)

**Expected behavior activated:**

- ”Spontaneously” approach a pedestrian in the established area, activating a genuine behavior, in the following steps:
  - A) Salute she/he: ”Hello. I’m from / I’m coming from … (it is mentioned the city / see below)

[in Romanian: *“Bună ziua. Sunt / vin din....* (este menționată localitatea / vezi lista de mai jos)*] [Timing: around 5 seconds]

- - - Observation: you have to use linguistic cues / a distinctive regional accent for each experimental condition (see the list bellow / condition**)
  - B) Express (using a non-verbal behavior) your uncertainty and suggesting that you are “lost”. Just take out your right hand from your pocket with a small block-notes where it is “supposed to be” relevant information (the “name of the street you are looking for”). *Do not “force” in any way the interaction with the pedestrian. Only suggest your unrest. If your interlocutor will accept or not the interaction with you and your suggested non-verbally request for help, it is only up to she/he. We are only interested in this step to know if your interlocutor expresses her/his availability to help by stopping and by listening your request.* [Timing: around 5 seconds]

IF (only IF she/he is stopping and listening to you…)

- - C) Adress she/he your concrete request by asking for direction, using the following statement (suggesting her/him that you have just read the name of the street from your block-notes): “…I’m looking for the *Ioan Vodă (cel Cumplit)* Street. Could you direct me to get to it?” [in Romanian: “*Caut strada Ioana Vodă (cel Cumplit). Îmi puteți arăta cum să o găsesc?/Îmi puteți arăta cum să dau de ea?)*].

[Timing: around 10 seconds]

Behavioral indication: do not press in any way your interlocutor. Wait for her/his response, evidencing the same state of uncertainty. Listen carefully and wholly committed her/his response.

Immediately after her/his answer [Timing: no more than 10 seconds after], whatever she/his replies …

- - D) Address the next question: ”And please, could you also show me how to get to the *Catedrala*?” [in Romanian: *“De asemenea/…Și îmi aratați cum să ajung la Catedrală?*]

Listen carefully and committed to her/his indications until the end (if she/he provides them) and…(if she/he offers them to you)…

- - E) Thank and politely salute she/he [in Romanian: *“Mulțumesc. O zi bună*”].

(this is the end of your role)

**Observations:**

*The list of the cities for each experimental condition (1/region):

| C1 | Buziaș |
| --- | --- |
| C2 | Brad |
| C3 | Băilești |
| C4 | Giurgiu |
| C5 | Vaslui |

**The list of linguistic cues/accent marks:

(only in Romanian)

| C1 | Bună ziua. *Mi-s* din Buziaș... |
| --- | --- |
| C2 | Bună ziua. *Îs* din Brad… |
| C3 | Bună ziua. *Venii* din Băilești… |
| C4 | Bună ziua. Sunt d*î*n Giurgiu… |
| C5 | Bună z*î*ua. *I-os* din Vaslui… |

**Selection procedure:**

- The general rule: in each trial you have to select each 10^th^ pedestrian you are counting from the beginning of a new trial, in the designated area (decided before with the coordinator of the study).
- If one of she/he is not fitting with the selection criteria, you have to select the next one.

Criteria for selection (how you *exclude* a pedestrian for selection):

- *Do not* select a pedestrian if…
  - she/he is:
    - in a hurry;
    - expresses any explicit distress;
    - not alone;
    - involved in another task (like reading something on her/his mobile, or talking on the mobile-phone and so one...).
